# Educating and Empowering Inner-City High School Students in Bleeding Control

**DOI:** 10.5811/westjem.2021.12.52581

**Published:** 2022-02-14

**Authors:** Millicent Okereke, Jessica Zerzan, Elizabeth Fruchter, Valerie Pallos, Maya Seegers, Mehr Quereshi, Lynn Model, Monique Jenkins, Gia Ramsey, Christine Rizkalla

**Affiliations:** Maimonides Medical Center, Department of Emergency Medicine, Brooklyn, New York

## Abstract

**Introduction:**

Unintentional bleeding is the leading cause of death in people 1–44 years of age in the United States. The Stop the Bleed (STB) campaign is a nationwide course that teaches the public to ensure their own safety, call 911, find the bleeding injury, and achieve temporary hemorrhage control by several techniques. Although the national campaign for the training course was inspired by active shooter events, the training can be applied to motor vehicle accidents and small-scale penetrating and gunshot wounds. Extending the audience to inner-city high school students in a violence-prone neighborhood has the potential to save lives if they are first on the scene.

**Objectives:**

We hypothesized that students would have a greater degree of comfort, willingness, and preparedness to intervene in acute bleeding after taking the course.

**Methods:**

This was a prospective, interventional pilot study in one inner-city high school in Brooklyn, New York. Students were given the option to participate in the STB course with pre- and post-surveys. We recruited 286 students from physical education or health education class to take a 50-minute bleeding control training course. Mean age was 15.7 years old. Students were divided into groups of 20–25 and taught by 2–3 emergency medicine, pediatric, or trauma surgery STB instructors. Each course included 2–3 skills stations for placing a tourniquet, wound packing, and pressure control.

**Results:**

Prior to the course, only 43.8% of the students reported being somewhat likely or very likely to help an injured person who was bleeding. After the course, this increased to 80.8% of students even if no bleeding control kit was available. Additionally, there were significant improvements in self-rated comfort level from pre- to post-course 45.4% to 76.5%, and in self-rated preparedness from 25.1% to 83.8%. All three measures showed statistically significant improvement, P <.0001.

**Conclusion:**

Teaching the STB course to high school students from a community with high levels of violence resulted in increased comfort level, willingness, and preparedness to act to control bleeding. If these opinions translate into action, students’ willingness to act could decrease pre-hospital blood loss and empower youth to perform life-saving interventions.

## INTRODUCTION

Unintentional and uncontrolled bleeding is the leading cause of death in people ages 1–44 in the United States. Per the US Centers for Disease Control and Prevention, trauma, most commonly traumatic brain injury and uncontrolled hemorrhaging, is the leading cause of death among pediatric populations. Exsanguinating hemorrhage can lead to death within a matter of minutes, making prompt hemorrhage control imperative in bleeding scenarios.^1^ In 2015 the National Trauma Institute estimated that severe bleeding accounts for greater than 35% of prehospital deaths and 80% of mass casualty victims are transported to the hospital by non-ambulance.^2^ Therefore, in the minutes before emergency medical technicians (EMT) can arrive to the scene, non-medical bystanders who are trained in basic techniques to control bleeding can be active participants in decreasing rates of preventable deaths.^3^

The Stop the Bleed (STB) campaign was started in 2013 to reduce the morbidity and mortality associated with hemorrhage secondary to traumatic injury.^4^ While STB initially developed from the US military, it has emerged as a major public health initiative.^5^ By 2015 the Department of Homeland Security launched a nationwide STB campaign to teach bystanders hemorrhage control. The STB training course, called Bleeding Control or B-Con, teaches the public to ensure their own safety, call 911, find the bleeding injury, and compress (either by covering the wound/direct pressure or using a tourniquet). The STB course can motivate participants by teaching them how to distinguish between life-threatening and non-life-threatening hemorrhage, and then slow it down.^6^ At its inception, the STB campaign had two main goals: 1) to inform as well as empower laypersons to be trained in basic trauma care to stop or slow bleeding during an emergency; and 2) to increase bystanders’ access to bleeding control kits. Using these skills, participants may be more likely to intervene in a time of need.^1^

While these techniques were initially used in mass casualty settings such as warfare, the national campaign for STB training course was inspired by the Sandie Hook elementary school shooting. The utility of these skills in other scenarios or injuries causing massive hemorrhage, such as motor vehicle accidents or penetrating wounds, has been increasingly recognized.^1^ Hemorrhage control techniques have the potential to be readily acquired by teenagers and could be particularly applicable in violence-prone areas with high rates of neighborhood shootings and stabbings.^7^

We believe that inner-city high school students can learn techniques to intervene in a bleeding injury if a family member, friend, or community member is involved in a violent event. The specific aim of this investigation was to teach bleeding control to high school students from the Brownsville neighborhood of Brooklyn and assess whether it increased their comfort level, willingness, and preparedness to intervene in hemorrhage control after taking part in the STB training course. In this investigation we trained the students in bleeding control techniques to empower them to use these skills before EMTs arrive.

**Population Health Research Capsule**

What do we already know about this issue?

*Stop the Bleed (STB) training prepares the public to save lives by teaching them bleeding control measures.*

What was the research question?

*Can students increase their comfort level, willingness, and preparedness to intervene after taking a bleeding control training course?*

What was the major finding of the study?

*Students had an increase in comfort level, willingness, and preparedness to intervene in controlling life-threatening bleeding.*

How does this improve population health?

*High school students trained in STB can help save lives before paramedics arrive by performing bleeding control measures.*

## METHODS

### Study Design and Setting

We chose to extend training to high school students in Brownsville because of the potential exposure to and crucial implications for survival of violent incidents. The neighborhood’s poverty rate is nearly double that of New York City and is the second highest in the borough of Brooklyn. Nearly 40% of Brownsville’s residents live below the federal poverty level compared to 21% in New York City.^8^ Brownsville ranks highest compared to other areas of New York City in the injury assault rate and non-fatal assault-related hospitalizations (180 per 100,000 population compared to 59 per 100,000 in other parts of New York City).^9^ Nearly three-quarters of the Brownsville section of Brooklyn is Black, and these neighborhoods have suffered disproportionately from a history of racial injustice, inequality, and poverty.^8^ From 2013–2017 it had the city’s highest homicide rate, with 16.9 deaths for every 1000 residents, four times the New York City average of 3.8 per 1000.^9^ With this disproportionate exposure to violence, our subjects are more likely to encounter a bleeding individual.

This was a prospective, interventional pilot study performed by physicians from the departments of emergency medicine and trauma surgery of an inner-city medical center in Brooklyn, New York. Investigators had prior exposure to STB in an introductory course during their medical training; however, for this study each physician successfully completed a STB instructor course to receive certification to teach participants. The investigation was conducted at a charter high school located in the Brownsville section of eastern Brooklyn during January–March 2020. In 2016 the Community Health Profile ranked the Brownsville section of Brooklyn, NY, fourth of 59 community districts across New York City in overall risk, including economic security, education, housing, health, youth issues, and violence, making it one of the highest overall risk-prone communities.^10^ All participation was voluntary and pre- and post-course paper surveys were anonymous. Investigators provided information about the study to students and parents, including the option to opt out without influence on school grades. The eligibility criteria included students ages 13–19. There were no exclusions, including prior training in a STB course. Approval for the study was obtained from our institutional review board.

### Data Collection and Analysis

The STB instructors distributed and collected data in a paper survey format before and after the student training course. There is no standard or validated tool from the STB campaign to assess participants’ attitudes regarding comfort or likelihood to intervene with a bleeding victim; therefore, the pilot pre- and post-course surveys were created by study investigators. Survey data was summarized as proportions. We compared the students’ likelihood/willingness, comfort, and preparedness before and after the training course was implemented. Students who were more likely, comfortable, and prepared to help an injured person were categorized as *positive,* and those who were more worried about causing further harm to an injured person were cartegorized as *negative*. We performed statistical analysis using the McNemar test, with statistical significance set to *P* < 0.05. The McNemar test, which is used to analyze categorical data in surveys/ questionnaires, determines differences on a dichotomous dependent variable between two related groups.

#### Self-Reported Inclination

We assessed participants’ attitudes and inclination to intervene in caring for victims experiencing severe bleeding secondary to traumatic injury in terms of their self-reported comfort level, willingness, and preparedness.

#### Procedure

We trained high school students on tourniquet placement, wound packing, and pressure application. The training courses were held during physical education or health education class throughout the school day. Participation in the program was optional, with an alternate assignment/activity provided by their teacher during that class period. Students completed a seven-item pre- and post-intervention anonymous paper survey and rated their experiences on a five-point Likert scale.

The training course and survey took the duration of the 55-minute class period. Each class included 25–30 students taught by 2–3 STB instructors. Courses included a 20–25 minute STB interactive PowerPoint, with time allotted for discussion, followed by a 15-minute skills station in which students were divided into three equal groups. One course instructor led each skills group, teaching hands-on practice with tourniquet placement, wound packing, and pressure control. At the end of the training, we provided students an eight-item post-intervention, anonymous, paper survey to complete before the class period was over. The additional question added to the post-survey concerned student apprehension or willingness to help a bleeding victim. A STB certificate was given to each student upon completion of their training.

## RESULTS

We recruited 290 high school students to participate in the pilot study, and 286 of those students chose to take part. In total, 98.6% (40.8% female) of the recruited students completed surveys and participated in the course. Mean age (±standard deviation [SD] was 15.7 years old ±1.2). As per Table 1, prior to the course only 43.8% of the students were somewhat likely or very likely to help an injured person who was bleeding. After the course, this increased to 80.8% of students even if no bleeding control kit was available. Additionally, there were significant improvements in self-related comfort level from pre- to post-course – 45.4% to 76.5% – and in self-rated preparedness from 25.1% to 83.8%. All three measures (willingness, comfort level, and preparedness) showed statistically significant improvement (*P* <.0001). As per Table 2, students post-STB course demonstrated a change in their likelihood, comfort level, and preparedness to help a bleeding person. Pre- and post-course surveys showed the following: 79.6% of students became somewhat to very likely to render aid; 63.1% were somewhat to very comfortable; and 81.2% were somewhat to very prepared. It should be noted that after the STB course, 6.8% of students reported they were less likely to intervene in helping a bleeding person, 8.8% less comfortable, and 8.9% less prepared.

**Figure 1:**
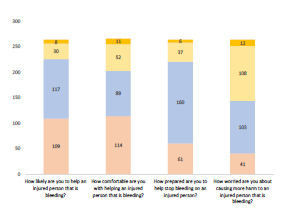
Pre-post intervention demonstrating changes in comfort level and willingness to render aid.

**Figure 2:**
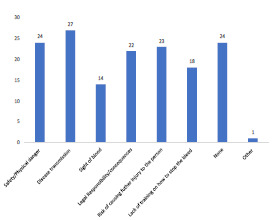
Self-reported concerns.

As per Figure 1, following the STB training course, students showed a statistically significant improvement (*P* <.0001) in attitude from pre- to post-surveys in their likelihood, comfort, and preparedness to help a bleeding person. The greatest concern students reported in bleeding intervention following the training course was disease transmission, followed by a concern about their safety or threat of physical danger (Figure 2). Subjects were allowed to choose more than one concern, or as many as applicable.

## DISCUSSION

In this study, high school students who participated in the STB training course were shown to have an increase in their comfort level, willingness, and preparedness to intervene in controlling life-threatening bleeding. While the STB training course has not been specifically validated in the adolescent population, popular media has reported on the ease of course delivery to high school students.^11^ Goolsby et al previously examined high school students’ ability to learn hemorrhage control skills and knowledge using the STB training course. This is the first study to demonstrate that high school students can learn hemorrhage control via multiple educational modalities: instructor-led; web-only; or a combination were assessed. Results showed all modalities improved participants’ self-reported willingness and comfort in using tourniquets.^12^ To the best of our knowledge, this is the only study to assess whether the STB training course increases the comfort level, willingness, and preparedness of inner-city students in particular. A similar study by Nanassy et al showed an increase in perceptions of self-efficacy and preparedness of school personnel at an urban high school in responding to a life-threatening bleeding emergency after completing a STB training course. 13 The Nanassy study included school personnel, while our study involved high school students; however, the similar findings of increased preparedness lend validity to the importance of STB training.

Since the course was established to teach the layperson without specifying an age group or reading level, bleeding control is, therefore, a skill that both teenagers and adults can learn to prevent further blood loss, and may be more crucial in communities with higher rates of violence. The results from pre-course to post-course surveys revealed a significant change in the participants’ willingness and comfort level in helping others. Thematic analysis of self-reported concerns about helping to stop active bleeding revealed that participants were more concerned about personal safety or physical danger and disease transmission to themselves when it came to helping a bleeding victim. We believe these findings have important implications for public health. Our results show that with a single STB course, high school students become more willing and more comfortable helping others.

## LIMITATIONS

This investigation was a small pilot study in a single environment, which may not be representative of other communities. It was conducted in a space that may not have been optimized for proper instruction; the health education classroom used was smaller than the physical education gymnasium, which was larger and potentially more conducive to hands-on, multi-group, and skills station instruction. The number of participants and classroom environments were not standardized. Of note, 11.4% of participants had prior training in a STB course, the context/location of which was not ascertained.

This pilot study assessed the efficacy of an educational intervention from self-reported survey responses; therefore, the impact may be limited due to its very design. However, it is an essential first step in assessing the potential benefits of this public health intervention. Our findings have implications for future research, practice, and education. We are unaware of whether comfort with bleeding control gained after this course will be maintained over time. In the study by Nanassy et al, thematic analysis of written responses showed that participants desired higher frequencies of STB training, more equipment, clearer school procedures, and realistic training scenarios with students.^13^ Similar investigations can be applied to studies in the future, such as follow-up surveys six months to a year after the course to assess retention and application in a real-life scenario. In addition to the follow-up surveys, focus groups and qualitative reporting on students’ experiences may lead to adjustments to the STB course, making the training better tailored for the age/population at hand. This was initially intended to occur after the completion of this pilot study in March 2020; however, the COVID-19 pandemic limited accessibility to the students.

## CONCLUSION

Teaching the Stop the Bleed course to inner-city high school students from a community with high levels of violence resulted in increased comfort, willingness, and preparedness to act to control bleeding in a victim. If these opinions translate into action, the students’ willingness to act could impact clinical practice and outcomes for EMTs and emergency physicians by decreasing prehospital blood loss and empower youth to perform life-saving interventions.

## Figures and Tables

**Table 1:** Demographics and surveys pre- and post-training in a Stop the Bleed course.

Question	Pre n (%)	Post n (%)
Age, mean(std)	15.7 (1.2)	
Gender, female	115 (40.8%)	
Prior Training	32 (11.4%)	
How likely are you to help an injured person that is bleeding?		
Not at all likely	22 (7.8%)	2 (0.7%)
Not likely	40 (14.2%)	5 (1.8%)
Not sure	96 (34.2%)	31 (10.8%)
Somewhat likely	86 (30.6%)	97 (33.9%)
Very likely	37 (13.2%)	134 (46.9%)
How comfortable are you with helping an injured person that is bleeding?		
Not at all comfortable	23 (8.1%)	5 (1.9%)
Not comfortable	51 (18.0%)	15 (5.6%)
Not sure	81 (28.5%)	43 (16.0%)
Somewhat comfortable	99 (34.9%)	150 (56.0%)
Very comfortable	30 (10.6%)	55 (20.5%)
How prepared are you to help stop bleeding on an injured person?		
Not at all prepared	43 (15.2%)	2 (0.8%)
Not prepared	83 (29.3%)	8 (3%)
Not sure	86 (30.4%)	33 (12.4%)
Question	Pre n (%)	Post n (%)
Somewhat prepared	65 (23.0%)	127 (47.6%)
Very prepared	6 (2.1%)	97 (36.3%)
How worried are you about causing more harm to an injured person that is bleeding?		
Very worried	74 (26.2%)	19 (7.1%)
Somewhat worried	77 (27.2%)	43 (16.1%)
Not sure	75 (26.5%)	60 (22.5%)
Not worried	40 (14.1%)	113 (42.3%)
Not at all worried	17 (6.0%)	32 (12.0%)
After participating in Stop the Bleed training, how important do you feel it is to have bleeding control equipment available in your school building or other public places?		
Not at all important	n/a	1 (0.4%)
Not important	n/a	4 (1.5%)
Not sure	n/a	31 (11.7%)
Somewhat important	n/a	66 (24.9%)
Very important	n/a	163 (61.5%)
How likely would you be to help a bleeding person if you did not have a bleeding control kit available?		
Not at all likely	n/a	7 (2.7%)
Not likely	n/a	16 (6.1%)
Not sure	n/a	48 (18.2%)
Somewhat likely	n/a	138 (52.3%)
Very likely	n/a	55 (20.8%)
After participating in Stop the Bleed training, do you have any concerns about helping to stop the bleed on an injured person?		
Safety/physical danger	n/a	74 (27.7%)
Disease Transmission	n/a	79 (29.6%)
Sight of blood	n/a	50 (18.7%)
Legal Responsibility/Consequences	n/a	52 (19.5%)
Risk of causing further injury to the person	n/a	63 (23.6%)
Lack of training on how to stop the bleed	n/a	46 (17.2%)
None	n/a	60 (22.5%)
other	n/a	3 (1.1%)

*std,* standard deviation.

*n/a,* not applicable.

**Table 2:** Pre- to post-changes in attitude toward helping a bleeding victim.

Question	Change from negative to positive feelings	Change positive to negative feelings	p-value*
How likely are you to help an injured person that is bleeding?	Of the 147 people who answered not at all likely through not sure, 117 switched to somewhat to very likely	Of the 117 people who answered Somewhat to Very Likely, 8 switched to not at all likely through not sure.	<.0001
How comfortable are you with helping an injured person that is bleeding?	Of the 141 people who answered not at all comfortable through not sure, 89 switched to somewhat to very comfortable	Of the 125 people who answered somewhat to very comfortable, 11 switched to not at all comfortable through not sure.	<.0001
How prepared are you to help stop bleeding on an injured person?	Of the 197 people who answered not at all prepared through not sure, 160 switched to somewhat to very prepared	Of the 67 people who answered somewhat to very prepared, 6 swtiched to not at all through not sure.	<.0001
How worried are you about causing more harm to an injured person that is bleeding?	Of the 211 people who answered very worried to not sure, 103 switched to not worried or not at all worried	Of the 53 people who answered not worried or not at all worried, 12 switched to very worried through not sure.	<.0001

* McNemar’s Test
